# Comparison of the incidence of adverse reactions between herbal decoction and Chinese patent medicine combined with western medicine in treatment of COVID-19: A protocol for systematic review and meta-analysis

**DOI:** 10.1097/MD.0000000000032255

**Published:** 2022-12-09

**Authors:** Yunliang Zhang, Xuhao Li, Wenyan Yu

**Affiliations:** a Department of Pain, Zibo Central Hospital, Zibo, People’s Republic of China; b School of Acupuncture-Moxibustion and Tuina, Shandong University of Traditional Chinese Medicine, Jinan, People’s Republic of China.

**Keywords:** Chinese patent medicine, coronavirus disease 2019, herbal decoction, incidence of adverse reactions, protocol

## Abstract

Coronavirus disease 2019 (COVID-19) is an acute respiratory infectious disease that has led to millions of confirmed cases and deaths worldwide. Traditional Chinese Medicine plays an irreplaceable role in the treatment and prevention of epidemics in China. Western medicine improves clinical symptoms as well as bringing many adverse reactions. The combination of Chinese and western medicine can significantly improve the clinical symptoms and efficacy of patients. Currently, there is a lack of systematic reviews on the comparison of the incidence of adverse reactions between the difference treatments. We conducted this study to evaluate the comparison of the incidence of adverse reactions between herbal decoction and Chinese patent medicine combined with western medicine in treatment of COVID-19.

**Methods and analysis:**

Randomized controlled trials in 9 databases from December 2019 to September 2022, will be included, without language restrictions. Two independent researchers will screen and select studies, extract data, and evaluate the study quality. The Cochrane risk-of-bias tool for randomized controlled trials will be used to assess the risk of bias in the included studies. Statistical analyses will be conducted using Review Manager.

**Results:**

Our findings will compare the incidence of adverse reactions between herbal decoction and Chinese patent medicine combined with western medicine in treatment of COVID-19, which will be disseminated in a relevant conference and published in a peer-reviewed publication.

**Ethics and dissemination:**

This study will not include personal information. Ethical approval is not required for this study.

**PROSPERO registration number:**

CRD42022371001.

## 1. Introduction

The world was surprised by the rapid spread of coronavirus disease 2019 (COVID-19), leading to a pandemic, death, and post-COVID syndrome. The first case of COVID-19 emerged in China in late 2019^[1]^ and rapidly spread to other countries, leading the World Health Organization to declare a state of an international public health emergency.^[2–4]^ COVID-19 is caused by the coronavirus SARS-CoV-2.^[5]^ The clinical manifestations of COVID-19 appear to be broad, ranging from fever, cough, tiredness, loss of taste or smell, sore throat, headache, pain and discomfort, and diarrhea, to severe symptoms such as loss of speech, mobility or confusion, severe pneumonia, and even death.^[6]^ Public health emergencies not only cause damage to the material life of society, but also affect the mental health of the public and patients.^[7]^

Western medicine is mainly antiviral in the treatment of COVID-19. Oral western medicine alone will produce many adverse reactions, such as nausea, vomiting and anorexia, liver function damage, skin itching, abdominal pain and diarrhea. During the COVID-19 epidemic, Chinese medicine has been used as a complementary treatment in China and has played a huge role in the treatment of COVID-19. Studies have shown that Traditional Chinese Medicine (TCM) has unique advantages in enhancing immunity, improving clinical symptoms of COVID-19, reducing complications and improving quality of life.^[8,9]^ The combination of Chinese and western medicine can greatly reduce the side effects caused by simply taking western medicine. Chinese patent medicine Lianhua Qingwei has the functions of anti-virus, anti-bacteria, inhibiting respiratory inflammation, reducing fever, relieving cough and resolving phlegm, etc.^[10,11]^ It can also reduce the adverse reactions such as vomiting and diarrhea caused by chemical drugs, which to some extent makes up for the defects of Western medicine treatment, and has important clinical significance.^[12]^According to the TCM theory, herbal decoction belongs to syndrome differentiation treatment. For patients with complex symptoms, decoction has the advantages of flexible compatibility, paying attention to prescribing a prescription for one person, and strong pertinence, which can well reduce all kinds of adverse reactions.

However, whether Chinese medicine can significantly reduce the incidence of adverse reactions needs further studied. In this paper, herbal decoction combined with western medicine and Chinese patent medicine combined with western medicine were statistically analyzed, aiming to find out the reasons for the inconsistent incidence of adverse reactions between the two treatment methods and provide reference for clinical medication of COVID-19.

## 2. Methods

### 2.1. Study registration

This study has been registered with PROSPERO (International Prospective Register of Systematic Reviews), and the registration number is CRD42022371001. This protocol is reported based on the guidelines of the Preferred Reporting Items for Systematic Reviews and Meta-analyses Protocols (PRISMA-P) 2015.^[13]^ If amendments are required, we will update our protocol to include any changes in the entire research process.

### 2.2. Eligibility criteria

The PICOS principles will be consulted to establish the inclusion and exclusion criteria for this systematic review.

#### 2.2..1. Studies.

Randomized controlled trials on the combination of Chinese herbal and western medicine in the treatment of COVID-19 will be comprehensively searched without restrictions on language or publication date. Additionally, unpublished documents will be manually searched. The following will be excluded: reviews; experience summaries; retrospective trials; prescription statistics; case reports; animal research literature; literature without adverse reaction outcomes; trials in which the western medicine group and the combined Western and Chinese/Western medicine group combined other treatments; trials in which the combined Western and Chinese medicine group had patients taking both herbal decoction and Chinese patent medicine; trials that treat only one symptom of COVID-19; taking medicine before and after self-control trials; trials in which the literature indicated that the grouping was non-randomized.

#### 2.2..2. Participants.

Participants who have been assessed as COVID-19 will be included. There are no restrictions on gender, education, race, age, or disease stage.

#### 2.2..3. Interventions and comparisons.

Intervention: The Chinese and western medicine combination group was treated with decoction or Chinese patent medicine combined with western medicine.

Control: The western medicine group alone was treated with the same western medicine as the combination group.

#### 2.2..4. Outcomes.

The outcome index was the occurrence of adverse reactions.

### 2.3. Search strategy

According to the PICO principles of the Cochrane Manual for the Systematic Evaluation of Interventions (hereinafter referred to as “the Cochrane Manual”) [P: participants; I: interventions; C: comparisons O: outcomes] and the treatment protocols recommended in the “New Coronavirus Pneumonia Protocols (First to Eighth Editions),” combined with the clinical practice for the use of drugs for new coronavirus pneumonia, to set the search terms.

We will conduct our research in five English databases, including PubMed, Embase, Cochrane Library, Web of Science, Medline, and in four Chinese databases, including China National Knowledge Infrastructure, China Biology Medicine disc, Wanfang Database, and VIP Database. Using different databases, we will combine keywords and free words for a comprehensive search. Taking PubMed as an example, Table 1 presents the complete search strategy.

**Table 1:** PubMed search strategy.

Number	Search items
#1	“COVID 19” [Title/Abstract] or “2019-nCoV” [Title/Abstract] or “coronavirus disease 19” [Title/Abstract] or “coronavirus disease 2019” [Title/Abstract] or “disease 2019 coronavirus” [Title/Abstract] or “sars coronavirus 2 infection” [Title/Abstract] or “SARS-CoV-2” [Title/Abstract]
#2	“Chinese medicine” [Title/Abstract] OR “Chinese herbal” [Title/Abstract] OR “Chinese and Western medicine” [Title/Abstract] OR “decoction” [Title/Abstract] OR “Chinese patent medicine” [Title/Abstract] OR “clear lung detox soup” [Title/Abstract] OR “patchouli” [Title/Abstract] OR “golden flower clear sense of particles” [Title/Abstract] OR “Lianhua Qingwen” [Title/Abstract] OR “dredge wind and detoxify” [Title/Abstract] OR “Fanggeng Tongsheng” [Title/Abstract] OR “Xingnaojing injection” [Title/Abstract] OR “Shengmai injection”[Title/Abstract] OR “Tanreqing injection” [Title/Abstract] OR “Xuebijing injection” [Title/Abstract] OR “Reduning injection”[Title/Abstract] OR “Shenfu injection” [Title/Abstract] OR “Shenmai injection” [Title/Abstract] OR “Fanggeng Tongsheng” [Title/Abstract]
#3	“Western medicine” [Abstract] or “conventional treatment” [Abstract] or “antiviral drugs” [Abstract] or “interferon alpha” [Abstract] or “lopinavir” [Abstract] or “ribavirin” [Abstract] or “ritonavir” [Abstract] or “arbidol” [Abstract] or “oseltamivir” [Abstract] or “chloroquine phosphate” [Abstract]
#4	“adverse reactions” [Abstract] or “adverse event” [Abstract] or “safety” [Abstract]
#5	#1 and #2 and #3 and #4

### 2.4. Data collection and analysis

#### 2.4..1. Selection of studies.

**Figure 1.**
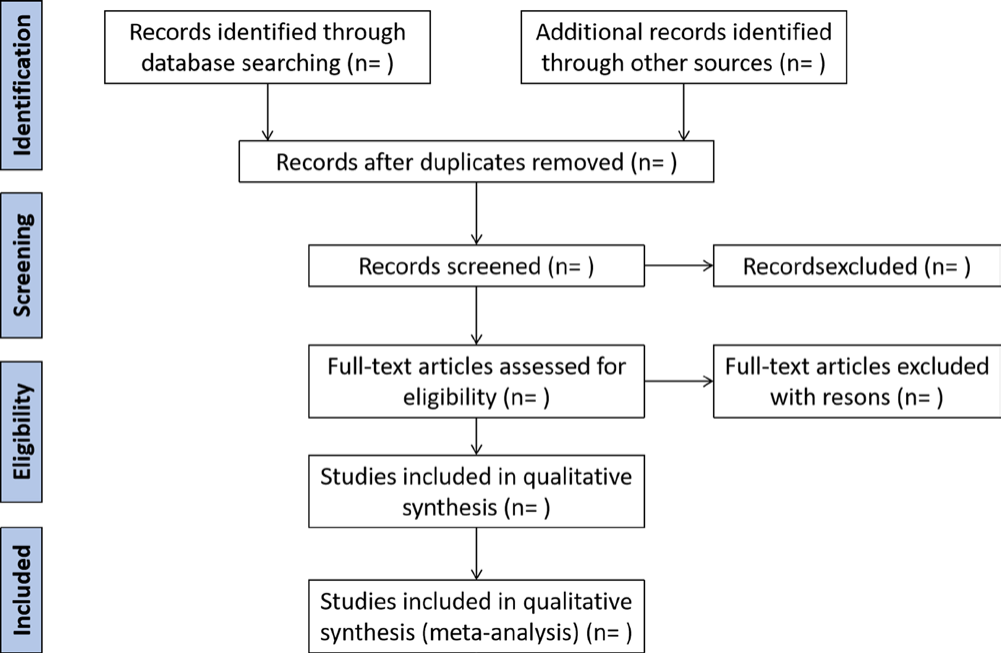
The PRISMA flow diagram. PRISMA = Preferred Reporting Items for Systematic Reviews and Meta-analyses.

The search strategy and study selection will be performed independently by 2 researchers (Y.L.Z. and L.X.H.), and the final research choices will be agreed upon. Two researchers independently evaluate the same article to determine their eligibility for inclusion and resolved differences through consensus, which will be managed by a third reviewer (Y.W.Y.). The selection process will be summarized using a PRISMA flow diagram. Details of the selection procedure for the studies are shown in the PRISMA flow chart (Fig. 1).

#### 2.4..2. Data extraction and management.

According to the Cochrane Handbook for Systematic Reviews of Interventions, the two researchers (Y.L.Z. and L.X.H.) will extract data, including the first author, year of publication, sample size, age, intervention measures, and number of adverse reactions. All database retrieval, literature screening and information extraction were carried out by two researchers in accordance with pre-set rules and standards. In case of any disagreement, a joint decision was made after discussion with a third party.

#### 2.4..3. Dealing with missing data.

If the information is missing or unclear, we will try to contact the corresponding author for more detailed information. If it fails, we will analyze it based on the available data.

#### 2.4..4. Assessment of risk of bias.

Two researchers (Y.L.Z. and L.X.H.) will assess the quality of the included studies independently by utilizing the Cochrane Risk of Bias assessment tool. As specified by the Cochrane Handbook (version 6.0), the following sources of bias will be considered: random sequence generation, allocation concealment, blinding of participants and personnel, blinding of outcome assessments, incomplete outcome data, selective reporting, and other sources of bias. Studies will be rated as having a high, low, or unclear risk of bias as appropriate.^[14]^ The rating results will be cross-checked, and discrepancies will be resolved through discussions and arbitration with a third author (Y.W.Y.).

#### 2.4..5. Assessment of quality of evidence.

The Grading of Recommendations Assessment, Development and Evaluation system will be used to assess the overall quality of the evidence derived from the included studies.^[15]^ In addition, the results will be divided into high, moderate, low, and very low quality.

#### 2.4..6. Measures of treatment effect.

Comparision of acupuncture efficacy will be performed by Review Manager software 5.4. For continuous results, the data are represented as the mean difference or standard mean difference with a 95% confidence interval. When dichotomous data were available, a hazard ratio with 95% confidence interval was used. When binary data exist, the RR format is changed for analysis.

#### 2.4..7. Heterogeneity evaluation.

Clinical and statistical heterogeneity between the studies will be assessed. Clinical heterogeneity will be assessed according to the similarity of research objects, intervention measures, control, and outcome indicators, while statistical heterogeneity will be evaluated by *I*^2^. If *I*^2^ ≤ 50% and *P* > .1, statistical homogeneity will be considered good. A fixed-effects model will be used for merging. If *I*^2^ > 50% or *P* ≤ .1, which indicates statistical heterogeneity, the source of heterogeneity will be further analyzed. A random-effects model will be used for meta-analysis after excluding obvious clinical heterogeneity. When there is obvious clinical heterogeneity, it should be treated by subgroup analysis, sensitivity analysis, or only descriptive analysis.

#### 2.4..8. Assessment of publication bias.

If more than 10 studies are included, we will use funnel plot and Egger’s test to assess the publication bias.^[16]^

#### 2.4..9. Data synthesis.

We take advantage of Review Manager software 5.4 for data analysis and synthesis. The relative risk and 95% confidence interval were used to analyze the effect values, and the *P* value and *I*^2^ were used to judge the heterogeneity. When *P* > .1 and *I*^2^ ≤ 50%, homogeneity among studies is considered, and the fixed-effects model will be used. When *P* ≤ .1 and *I*^2^ > 50%, it was considered that heterogeneity existed among the studies. Therefore, a random-effects model will be used for analysis, and sensitivity analysis method was used to speculate the source of heterogeneity. Subgroup or sensitivity analyses will be performed, if there is significant clinical heterogeneity. *P* < .05 was considered statistically significant. Funnel plots were drawn and analyzed for more than 10 literatures.

#### 2.4..10. Subgroup analysis.

If the heterogeneity is high, we will also perform subgroup analysis to calculate the combined statistics.^[14]^ The following subgroup analyses will be considered: gender, age, intervention time, intervention cycle, and course of the disease.

#### 2.4..11. Sensitivity analysis.

Sensitivity analysis will be performed to investigate the robustness of the primary outcomes, which includes assessing the method quality, study quality, and impact of sample size and missing data.

#### 2.4..12. Ethics and dissemination.

Since this study will be a secondary analysis of primary studies, no patients or public participants will be involved, and no primary data will be collected. Therefore, ethical approval is not required for this study. The results of this study will be shared and presented in conference reports and peer-reviewed journals.

## 3. Discussion

This article is the first to analyze the effects of decoction and Chinese patent medicine combined with western medicine on the incidence of adverse reactions in the treatment of COVID-19. As an external treatment method of TCM, Chinese medicine has the characteristics of simple and simple verification. It can not only assist the treatment but can also greatly reduce the adverse effects.

This study provides explanations for the inconsistent results of numerous studies, as well as providing exploration directions for reducing the risk of COVID-19 treatment. We hope that this review will provide more convincing evidence for clinicians to treat these conditions and help them make appropriate decisions.

## Author contributions

All the authors had access to the data and played a role in writing the manuscript. All authors have read and approved the final manuscript.

**Data curation:** Yunliang Zhang.

**Formal analysis:** Xuhao Li.

**Methodology:** Wenyan Yu, Xuhao Li, Yunliang Zhang.

**Project administration:** Wenyan Yu.

**Resources:** Yunliang Zhang.

**Software:** Yunliang Zhang.

**Visualization:** Xuhao Li.

**Writing – original draft:** Yunliang Zhang.

**Writing – review & editing:** Wenyan Yu.
